# Miniature- and Multiple-Eyespot Loci in *Chlamydomonas reinhardtii* Define New Modulators of Eyespot Photoreception and Assembly

**DOI:** 10.1534/g3.111.000679

**Published:** 2011-11-01

**Authors:** Joseph S. Boyd, Mary Rose Lamb, Carol L. Dieckmann

**Affiliations:** * Department of Molecular and Cellular Biology, University of Arizona, Tucson, Arizona 85721; † Department of Biology, University of Puget Sound, Tacoma, Washington 98416

**Keywords:** eyespot, photoreception, organelle biogenesis, *MIN2*, *MLT2*

## Abstract

The photosensory eyespot of the green alga *Chlamydomonas reinhardtii* is a model system for the study of organelle biogenesis and placement. Eyespot assembly and positioning are governed by several genetic loci that have been identified in forward genetic screens for phototaxis-defective mutants. These include the previously described miniature-eyespot mutant *min1*, the multiple-eyespot mutant *mlt1*, the eyeless mutants *eye2* and *eye3*, and two previously uncharacterized eyespot mutants, *min2* and *mlt2*. In this study, effects of miniature- and multiple-eyespot mutations and their combinations on the localization and expression levels of the rhodopsin photoreceptor channelrhodopsin-1 (ChR1) and the localization of the eyespot-assembly proteins EYE2 and EYE3 were examined. *min2* mutants assemble a properly organized, albeit nonfunctional, eyespot that is slightly smaller than wild-type; however, combination of the *min2* and *mlt1* mutations resulted in drastic reduction of photoreceptor levels. Both stationary-phase *mlt1* and *mlt2* cells have supernumerary, mislocalized eyespots that exhibit partial or total dissociation of the eyespot layers. In these mutant strains, photoreceptor patches in the plasma membrane were never associated with pigment granule arrays in the chloroplast stroma unless EYE2 was present in the intervening envelope. The data suggest that MIN2 is required for the photoreceptive ability of the eyespot and that MLT2 plays a major role in regulating eyespot number, placement, and integrity.

Sensory organelles, such as the primary cilium, are specialized for detection of external stimuli and often occupy defined positions within the cell to facilitate specific cellular responses (Pazour and Witman 2003; Bornens 2008). The eyespot of the biflagellate, unicellular green alga *Chlamydomonas reinhardtii* is an asymmetrically localized, photosensory organelle that mediates directional light perception and allows the cell to respond to varying light levels by either swimming toward a source of low-intensity light (positive phototaxis) or away from high-intensity light (negative phototaxis) (Witman 1993; Kreimer 1994, Hegemann 1997). Light-induced plasma membrane depolarization is sensed by Ca^2+^-responsive proteins, eliciting changes in the flagellar beat pattern and swimming orientation (Nultsch 1983; Kamiya and Witman 1984; Hegemann *et al.* 1990). The eyespot of *C. reinhardtii* brings together multiple cellular compartments, comprising components in both the chloroplast and plasma membrane as well as maintaining a characteristic association with the cytoplasmic cytoskeletal system. The eyespot, which in wild-type *C. reinhardtii* cells has an average diameter of one micrometer (Harris 1989), resides in a defined position in the cell in association with the daughter four-membered (D4) microtubule rootlet and is situated 45° from the plane of the flagella (Holmes and Dutcher 1989), an arrangement necessary for mediating proper photoresponses (Foster and Smyth 1980; Kamiya and Witman 1984). The *C. reinhardtii* eyespot is not inherited from the mother cell but forms anew each cell cycle (Holmes and Dutcher 1989). The eyespot comprises an average of 120 carotenoid-filled pigment granules (Melkonian and Robenek 1984) arranged in two to four layers in the chloroplast. The pigment granule arrays are stacked between layers of thylakoid membrane and are tightly apposed to the chloroplast envelope (Melkonian and Robenek 1984; Dieckmann 2003; Kreimer 2009) and appear as a distinct spot when viewed in the light microscope (see Figure 1A).

**Figure1:**
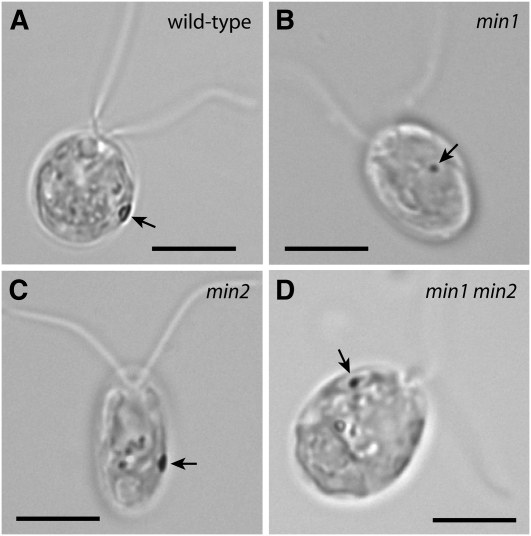
Phenotypic characterization of *Chlamydomonas reinhardtii* miniature-eyespot mutants. (A–D) Bright field micrographs. Arrows indicate eyespots. Bars, 5 μm. (A) Wild-type cell. (B) The *min1* mutant has a miniature, equatorially localized eyespot. (C) The eyespot of *min2* cells is equatorially localized and slightly smaller than wild-type. (D) *min1 min2* double-mutant cell. Over half of the cells of this mutant strain have no eyespot observable by bright field light microscopy. Eyespots of the remainder of cells in the population range in size from ultramini to approximately *min1*-sized.

The membranes in the eyespot region are highly specialized. The outer chloroplast envelope membrane is characterized by a high particle density observed by electron microscopy (Melkonian and Robenek 1980). Directly overlaying the pigment granule compartment is a particle-dense region of plasma membrane containing light-gated rhodopsin photoreceptors, channelrhodopsins 1 and 2 (ChR1 and ChR2) (Sineshchekov *et al.* 2002; Nagel *et al.* 2002; Nagel *et al.* 2003). The D4 rootlet is postulated to guide the photoreceptors to the site of eyespot assembly (Mittelmeier *et al.* 2011), where they form and maintain a stable association with eyespot proteins in the chloroplast envelope (Boyd *et al.* 2011a).

Unraveling the mechanisms that unify disparate cellular elements into a single functional system such as the eyespot is a formidable challenge that has been assisted by forward genetic approaches. Mutations in *C. reinhardtii* have identified several loci required for eyespot biogenesis, structure, and positioning, including *MIN1*, *EYE2*, *EYE3*, and *MLT1* (Lamb *et al.* 1999). Both the *eye2* and *eye3* mutants lack eyespots and are unable to phototax at low-light intensity. EYE2, a thioredoxin-family protein, localizes to the chloroplast envelope compartment of the eyespot, likely directing the site for assembly of the eyespot pigment granule arrays (Boyd *et al.* 2011a). The EYE3 protein is a ser/thr kinase of the ABC1 family localized to the eyespot pigment granules, and it is required for the biogenesis or stability of the granules (Boyd *et al.* 2011a). The *min1* mutant possesses a miniature eyespot characterized by disorganized and nonmembrane-apposed pigment granules in the chloroplast stroma when grown in medium lacking acetate as a carbon source (Lamb *et al.* 1999; Boyd *et al.* 2011a). MIN1 is a C2/LysM-domain protein (Mittelmeier *et al.* 2008) present in the eyespot proteome (Schmidt *et al.* 2006). The *mlt1* mutant has multiple eyespots that can form in either longitudinal hemisphere of the cell. The pigment granule arrays in eyespots of the *mlt1* mutant appear structurally normal by electron microscopy (Lamb *et al.* 1999). The putative MLT1 protein is not predicted to possess a chloroplast-targeting sequence and has no functional domains or homology to other proteins in the databases (Boyd *et al.* 2011b). Although previous studies have revealed much information regarding the factors that govern the coordination of the eyespot-assembly process and regulate the structural aspects of eyespot formation, many remain to be identified. Here we report the characterization of two additional eyespot mutants, the miniature-eyespot mutant *min2* and the multiple-eyespot mutant *mlt2*. Both the *MIN2* and *MLT2* loci map to existing clusters of eyespot-assembly genes and define novel factors that indicate the existence and integration of multiple processes in eyespot assembly and photoreceptive function.

## Materials and Methods

### Chlamydomonas strains and media

Chlamydomonas strains used in this study are listed in Table 1. *Chlamydomonas reinhardtii* wild-type strains 137c mt^+^ (CC-125) and mt^−^ (CC-124), were obtained from the Chlamydomonas Stock Center (University of Minnesota, St. Paul, MN). Strains *min1-1* (CC-4305) and *mlt1-1* (CC-4304) were originally obtained following UV-mutagenesis of strain 137c mt^+^ (Lamb *et al.* 1999). Strain *min2-1* (59-1; CC-4318) was a spontaneous mutation isolated following mutagenesis of strain 137c mt^+^ with the *CRY-1* insertion (Nelson *et al.* 1994). The mutation in *min2-1* is unlinked to the insertion. Strains 33 (CC-4317) and *mlt2-1* (2-8; CC-4320) were isolated following 5-fluorodeoxyuridine-induced mutagenesis of strain 137c mt^+^. Strains were maintained on solid tris-acetate-phosphate (TAP) medium or TAP supplemented with 0.2 mg/mL arginine (for arginine auxotrophic strains). Liquid cultures were grown in modified Sager and Granick medium I with added Hutner’s trace elements (R medium) or without acetate (M medium), or in M medium lacking nitrogen (M-N medium) (Harris 1989).

**Table 1:** Chlamydomonas strains used in this study

Strain	Genotype/Comments	Reference/Source
137c *mt^+^*	Wild-type	Harris (1989)
137c mt^−^	Wild-type	Harris (1989)
33	*eye3-3*	This study
59-1	*min2-1*	This study
12-10	*mlt1-1*	Lamb *et al.* (1999)
2-8	*mlt2-1*	This study
12-12	*min1-1* mt*^+^*	Lamb *et al.* (1999)

### Genetic screens and phototaxis assays

Mutagenized *Chlamydomonas reinhardtii* strains were screened using a simple assay for phototactic ability. Strains were patched on solid TAP medium plus arginine and inoculated into 1.2 mL liquid M-N medium in test tubes. Cultures were grown overnight at 25° and assayed for phototaxis by placement in a covered box with a narrow slit at the bottom for illumination. Phototaxis-defective (ptx^−^) or nonswimming strains were observed by bright field microscopy.

### Genetic analysis

Fresh cultures from plates were grown for two days on solid R medium containing one-tenth of the normal nitrogen source at 25° under continuous illumination. Cells were inoculated into 1 mL M-N medium, incubated four hours at 25°, and then 200 μL of each culture were combined and allowed to mate for one hour under continuous illumination at 25°. Mating mixtures were plated on solid R medium containing 4% agar and kept in the dark for at least four days. Dissection and tetrad analysis were conducted according to standard methods (Harris 1989). For complementation and dominance tests, eyespot mutant strains containing the *arg7-2* allele were mated to mutant strains containing the *arg7-8* allele. Mating mixtures were plated on solid TAP medium, without arginine to select for diploids, which were then assayed for phototactic ability as described above.

### Bright field microscopy

Cells from overnight liquid cultures were viewed according to the protocol described in Mittelmeier *et al.* (2008).

### Immunofluorescence microscopy

Preparation of samples and immunofluorescence microscopy were carried out according to the protocol described in Mittelmeier *et al.* (2008), except antibodies against EYE2, EYE3, and ChR1 were directly conjugated to fluorophores (Alexa Fluor 488, Alexa 594, or allophycocyanin) using Zenon rabbit IgG–labeling kits (Invitrogen, Carlsbad, CA) following the manufacturer’s protocol. Monoclonal anti–acetylated α-tubulin (Clone 6-11B-1, Sigma, St. Louis, MO) was detected with goat anti-mouse secondary antibodies conjugated to Alexa Fluor 568 or 647 at a dilution of 1:1000 or Cy5 (Molecular Probes) at a dilution of 1:200.

### Immunoblotting

Immunoblotting was carried out according to the protocol described in Mittelmeier *et al.* (2008), except primary antibodies were used at the following dilutions: 1:500 rabbit polyclonal anti-EYE2, 1:500 rabbit polyclonal anti-ChR1, and 1:10,000 mouse anti-tubulin (clone B-5-1-2; Sigma). Blots were probed with goat anti-rabbit horseradish peroxidase at a dilution of 1:5,000 and/or goat anti-mouse horseradish peroxidase (Pierce, Rockford, IL) at a dilution of 1:10,000 in 1% NFDM-TBS-T for 2 hr at room temperature. Protein levels were estimated from a digital image of the blot using the National Institutes of Health (NIH) ImageJ software Gel Anaylzer function. For each sample, the anti-ChR1 and anti-EYE2 signal were normalized to the antitubulin signal.

### Preparation of figures

Figures were prepared using Adobe Illustrator (Adobe Systems) and Microsoft Word (Microsoft). Micrographs were minimally adjusted for brightness and contrast using NIH ImageJ software, cropped in Adobe Photoshop, and reduced from the original size in Adobe Illustrator.

## Results

### *MIN2* is required for phototaxis and proper eyespot size

To expand the collection of known eyespot mutants, a genetic screen for strains defective in phototaxis was conducted following insertional mutagenesis of wild-type strain 137c mt^+^ with the *CRY-1* gene, which encodes ribosomal protein S14 (Nelson *Et Al*., 1994). A motile, ptx^−^ strain was isolated that exhibited an equatorially localized miniature eyespot by bright field microscopy (Figure 1C) and was named *min2-1*. The mutation in *min2-1* was found to be unlinked to the *CRY-1* insertion. The average area of eyespots measured in a *min2* population was 0.85 ± 0.16 μm^2^ (74% of wild-type area) compared with an average area of 0.38 ± 0.09 μm^2^ for *min1* cells (34% of wild-type area) and average area of wild-type eyespots of 1.2 ± 0.24 μm^2^ (Table 2).

**Table 2:** Eyespot area of miniature-eyed mutants

Strain	Average Eyespot Area (μm^2^)	SD (μm^2^)	% Wild-Type Area	*n*
Wild-type	1.2	0.24	100	100
*min1*	0.38	0.09	32	100
*min2*	0.85	0.16	71	100

**Figure 2:**
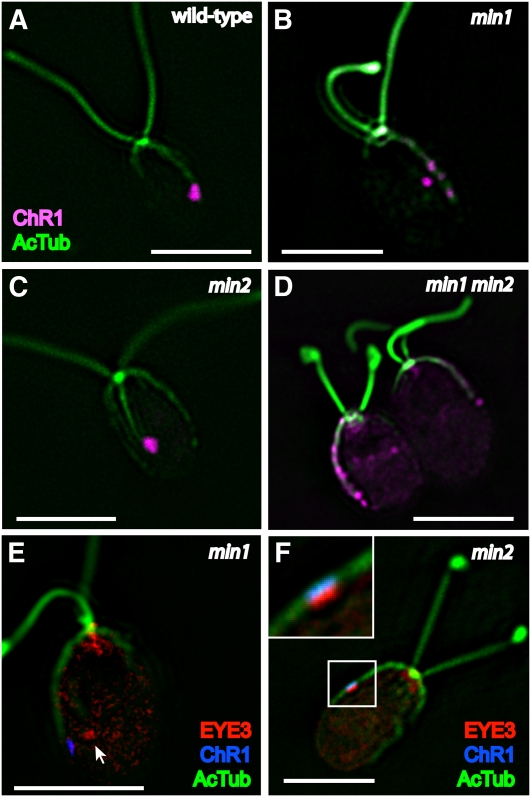
ChR1 photoreceptor localization and eyespot layers are altered in *min1* but not *min2*. (A–D) Combined immunofluorescence micrographs of fixed cells stained for channelrhodopsin-1 (ChR1, magenta) and acetylated α-tubulin (AcTub, green). (A) Wild-type cell with a ChR1 patch associated with the D4 microtubule rootlet. (B) ChR1 staining in *min1* cells appears as multiple, distinct spots or stripes along the D4 rootlet, occasionally appearing in off-rootlet spots. (C) The shape and position of the ChR1 patch on the D4 rootlet are maintained in *min2* mutant cells. (D) *min1 min2* cells, showing ChR1 staining in multiple spots along the D4 rootlet. (E–F) Combined immunofluorescence micrographs of fixed cells stained for the pigment granule marker EYE3 (red), ChR1 (blue), and AcTub (green). (E) Pigment granules (arrow) are not apposed to the plasma membrane-localized photoreceptor spots in photoautotrophically grown *min1* cells. (F) Organization of eyespot layers is unaffected in *min2* cells, with ChR1 directly overlaying EYE3 staining (inset). Scale bars, 5 μm.

As previously described, ChR1 photoreceptor localization is perturbed in the *min1* mutant, appearing as stripes or multiple spots along the D4 rootlet (Mittelmeier *et al.* 2008; Figure 2B). If MIN2 has a role in promotion of eyespot organization comparable to that of MIN1, similar ChR1 localization patterns might be expected to be observed in the *min2* strain. However, ChR1 staining patterns in *min2* cells double-stained with antibodies directed against ChR1 and acetylated tubulin were unaffected, the photoreceptor patch retaining its nearly wild-type elliptical shape and rootlet association (Figure 2C). *min1 min2* double-mutant cells were typified by multiple ChR1 patches associated with the rootlet (Figure 2D), a phenotype similar to that of the *min1* single mutant. When viewed by electron microscopy, the pigment granule spot in *min1* cells grown in medium lacking acetate as a carbon source is a disorganized aggregation in the chloroplast stroma that lacks apposition to the chloroplast envelope and plasma membranes (Lamb *et al.* 1999). This finding was corroborated by immunofluorescence staining for ChR1 and the pigment granule marker EYE3; in photoautotropically grown *min1* cells, the stromal pigment granules were separated from the photoreceptor (Figure 2E). By contrast, staining for both EYE3 and ChR1 in *min2* cells demonstrated that the photoreceptor patch directly overlayed pigment granule layers (Figure 2F). Thus, the *min2* mutation does not affect the overall assembly and/or maintenance of the eyespot layers.

### *min2* exacerbates the eyespot-assembly defect of *min1*

**Figure 3:**
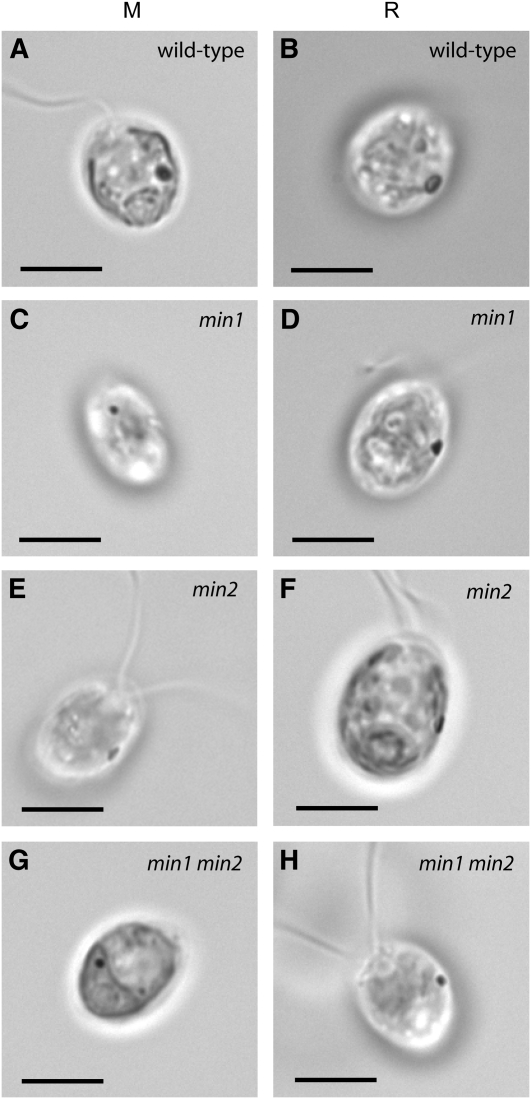
Comparison of eyespot morphology in wild-type, *min1*, *min2*, and *min1 min2* cells grown without acetate or with acetate. Cells shown in the left column were grown in M medium (no acetate) and cells in the right column in R (+ acetate). Whereas *min1* cells assemble a more organized, slightly larger eyespot when grown with acetate (D), no change in eyespot morphology is observed in wild-type (A, B), *min2* (E, F), or *min1 min2* cells (G, H). Scale bars, 5 µm.

*min1* mutant cells assemble a miniature, disorganized eyespot when grown in medium lacking acetate (Figure 1B), but *min1* cells grown in acetate-containing medium assemble eyespots that are more organized and closer in morphology to wild-type (Lamb *et al.* 1999; Mittelmeier *et al.* 2008). The eyespot morphology of *min2* did not appear to differ between cells from cultures grown photoautotrophically in M medium and cells from cultures grown mixotrophically in acetate-containing (R) medium (Figure 3, E and F). The combination of the *min1* and *min2* mutations resulted in cells with an intensified eyespot-assembly defect. Of *min1 min2* cells scored following overnight growth in M medium, 58% had no observable eyespot by bright field microscopy (Table 4). The remainder of cells in the population had a miniature eyespot that appeared to be approximately *min1*-sized (see Figure 1D). In addition, *min1 min2* cells grown mixotrophically were unable to assemble a larger eyespot (Figure 3, G and H). Thus, eyespots can assemble in the absence of both MIN1 and MIN2 function, but the lack of MIN2 function exacerbates the eyespot-assembly defect of *min1* mutants. The increased severity of the eyespot defects in *min1 min2* cells compared with those of *min1* cells is suggestive that MIN2 is needed for aspects of eyespot assembly distinct from those governed by MIN1.

### *MLT2* locus affects eyespot number, size, and placement

**Figure 4:**
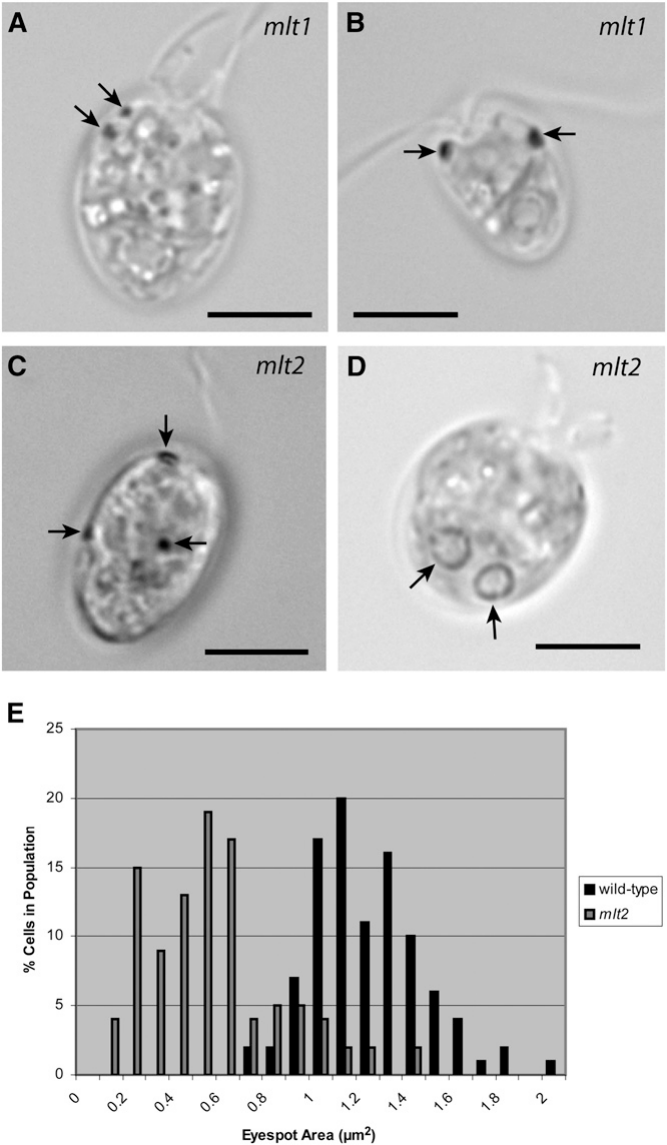
Characterization of multiple-eyespot mutant phenotypes. Arrows in A–C indicate eyespots. (A) *mlt1* cell with two eyespots on the same side of cell. (B) *mlt1* cell with an eyespot on both sides. (C) *mlt2* cell with three eyespots in various positions. Combined image from two focal planes. (D) Bright field micrograph of a *mlt2* cell with two pyrenoids (arrows). Two pyrenoids are present in 21% of *mlt2* cells, and 79% have one pyrenoid (n = 102). Bars, 5 µm. (E) Distribution of eyespot sizes in wild-type (n = 100 eyespots) and a *mlt2* population (n = 54 eyespots). Eyespots in the *mlt2* mutant are distributed over a wide size range but are, on average, smaller than wild-type.

In a separate genetic screen of approximately 200 strains for ptx^−^ mutants following 5-fluorodeoxyuridine-induced mutagenesis of wild-type strain 137c mt^+^, a strain that possessed multiple eyespots was identified and subsequently named *mlt2*. The *mlt2* mutation complements *mlt1-1* (10 ptx^+^ diploids with wild-type eyespots, n = 10). The *mlt2* mutant displays defects in eyespot size and positioning as well as misregulation of eyespot number. As observed by bright field light microscopy, *mlt2* cells have one to five eyespots that are positioned throughout the chloroplast (Figure 4C). Eyespots scored in a *mlt2* population ranged in size from 0.04 to 1.39 μm^2^ (Figure 4E) and were on average smaller than wild-type, with a mean area of 0.49 ± 0.29 μm^2^ (n = 54) compared with the typical wild-type average of 1.2 μm^2^ (strain 137c). Interestingly, 21% of *mlt2* cells have two pyrenoids (Figure 4D), but the strain had no observable growth defect (supporting information, Figure S1 and Table S1), suggestive that the multiple-pyrenoid phenomenon is not likely a result of defective cytokinesis. The *mlt2* mutation thus affects the number of a chloroplastic structure other than to the eyespot.

### Eyespot organization is perturbed in multiple-eyespot mutants

**Figure 5:**
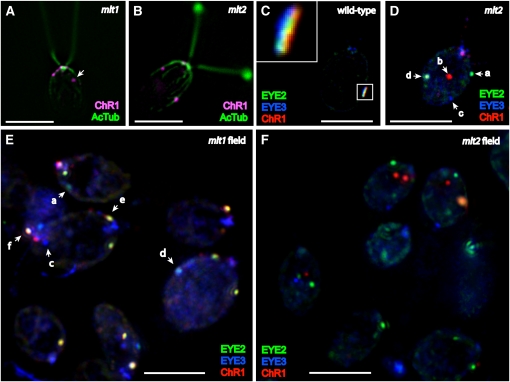
Eyespots are disorganized in *mlt1* and *mlt2* mutant cells. (A, B) Combined immunofluorescence micrographs of individual cells stained for ChR1 (magenta) and AcTub (green). (A) *mlt1* cells have ChR1 patches in either hemisphere of the cell that are often clustered around the anterior pole and associated with acetylated rootlets. Arrow indicates ChR1 patch not associated with a rootlet. (B) *mlt2* cell with multiple ChR1 patches associated with microtubule rootlets. (C) Wild-type cell showing layered arrangement of EYE2 (green), EYE3 (blue), and ChR1 (red) in the eyespot (inset). (D–F) EYE2, EYE3, and ChR1 positioning is dramatically disrupted in asynchronous stationary-phase populations of *mlt1* and *mlt2* cells. Combined immunofluorescence micrographs of fixed cells stained for EYE2 (green), EYE3 (blue), and ChR1 (red). a, single EYE2 spot; b:, single ChR1 spot; c, single EYE3 spot; d, EYE2/EYE3 copositioned spot; e, EYE2/ChR1 copositioned spot; f, EYE2/EYE3/ChR1 copositioned spot. (D) Z-projections of combined immunofluorescence micrographs of an individual *mlt2* cell illustrating positioning of EYE2, EYE3, and ChR1 spots. (E) Z-projection of combined immunofluorescence micrographs of a *mlt1* field stained for EYE2 (green), EYE3 (blue), and ChR1 (red). Various combinations of single and copositioned spots are observed (arrows). (F) Z-projection of combined immunofluorescence micrographs of a *mlt2* field. Various spot-positioning combinations are again observed, with single spots predominating. Scale bars, 5 µm.

**Figure 6:**
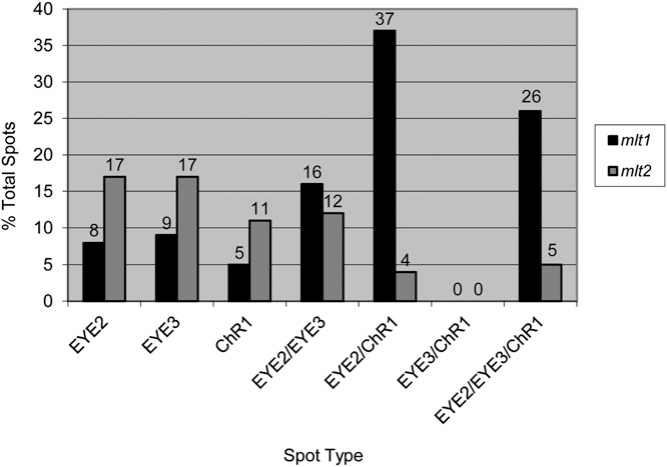
Quantification of the extent of EYE2, EYE3, and ChR1 copositioning in *mlt1* and *mlt2* cells. Percentage of single or copositioned spots per total spots scored is shown. Copositioned spots of combinations of EYE2, EYE3, and ChR1 are markedly more prevalent in *mlt1* cells compared with *mlt2* cells. Conversely, *mlt2* cells have a greater proportion of single EYE2, EYE3, or ChR1 spots not copositioned with other eyespot proteins. Copositioning of EYE3 with ChR1 was never observed without EYE2.

In the *mlt1* mutant, the ChR1 photoreceptor is localized to multiple patches most often observed in both longitudinal hemispheres of the cell in proximity to the anterior pole (Mittelmeier *et al.* 2011; see Figure 5A). Although ChR1 patches in *mlt1* cells are usually associated with a rootlet, some lack rootlet association (Figure 5A, arrow). Disjunction of ChR1 patches and pigment granule spots has also been observed in the *mlt1* mutant (Boyd *et al.* 2011b). Similar to the multiple eyespots observed in bright field, *mlt2* cells double-labeled for ChR1 and acetylated tubulin exhibited multiple ChR1 patches that were most often associated with a microtubule rootlet (Figure 5B). We examined whether the *mlt2* mutation elicits similar effects on the organization of eyespot components. In wild-type cells, distinct layering of ChR1, EYE2, and EYE3 is observed in the eyespot (Boyd *et al.* 2011a; Figure 5C). In *min1* and *eye3* mutants, in which the pigment granule layers are either disrupted or absent, EYE2 was most often observed copositioned with ChR1 (Boyd *et al.* 2011a). If the multiple-eyespot mutations predominantly affect the association of pigment granule layers with the rest of the eyespot, EYE2 might retain association with ChR1. In actuality, *mlt1* and *mlt2* were found to have dramatic effects on organization of eyespot components. In both *mlt1* and *mlt2* cells grown to stationary phase (∼1.0 × 10^7^ cells/mL), EYE2, EYE3, and ChR1 spots were visible singly (*i.e.* without either of the other two proteins associated) and in various combinations of copositioned spots (Figure 5F), including copositioning of EYE2 and EYE3 without ChR1. The distance between single spots was highly variable and could be relatively large in some cells (Figure 5D). Strikingly, copositioning of EYE3 and ChR1 was never observed in *mlt1* or *mlt2* cells unless EYE2 was also present in the copositioned spot (Table 3 and Figure 6), demonstrating that EYE2 is required for the chloroplast envelope link between the plasma membrane and pigment granule layers in the eyespot.

**Table 3:** Quantification of EYE2, EYE3, and ChR1 copositioning in asynchronous stationary-phase *mlt1* and *mlt2* populations

Strain	Spot Category	Single EYE2 Spot	Single EYE3 Spot	Single ChR1 Spot	EYE2/EYE3 Copositioned	EYE2/ChR1 Copositioned	EYE3/ChR1 Copositioned	EYE2/EYE3/ChR1 Copositioned
*mlt1*	% total single spots	39	40	21	–	–	–	–
	% total copositioned spots	–	–	–	20	47	0	33
	% total spots	8	9	5	16	37	0	26
*mlt2*	% total single spots	62	40	28	–	–	–	–
	% total copositioned spots	–	–	–	44	19	0	22
	% total spots	17	17	11	12	4	0	5

The extent of EYE2, EYE3, and ChR1 copositioning was distributed variably in populations of both *mlt1* and *mlt2* cells (Table 3); however, *mlt1* cells had a greater proportion of copositioned spots, while single spots of all three proteins predominated in stationary-phase *mlt2* cells (Figure 6). Eyespots in *mlt2* cells did show a large degree of copositioning in logarithmic growth phase (approx. 8.3 × 10^5^ cells/mL), with 77% of spots consisting of EYE2, EYE3, and ChR1 that were mutually copositioned. At this cell density, 32% of *mlt2* cells had one pigment granule spot, 55% had two pigment granule spots, and 13% had three or more pigment granule spots (n = 166). Together, these data are suggestive that to some extent MLT1, and especially MLT2, play roles in limiting formation of supernumerary eyespots and are required for maintenance of the supramolecular organization of the eyespot and that in the absence of *MLT1* or *MLT2* gene function, disintegration of the eyespot structure can occur after biogenesis of the organelle.

### Miniature-eyespot mutations suppress the multiple-eyespot defect of *mlt1*

**Figure 7:**
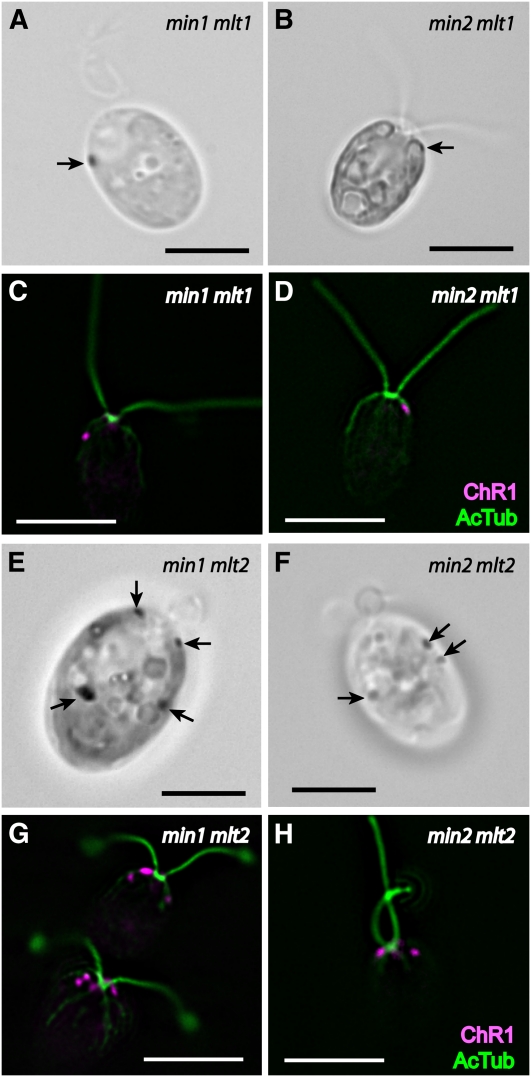
Miniature-eyespot loci affect eyespot formation and photoreceptor placement in combination with *mlt1* but not *mlt2*. (A, B) and (E, F) Bright field micrographs of individual cells. Arrows indicate eyespots. (C, D) and (G, H) Combined immunofluorescence micrographs of cells stained for ChR1 (magenta) and AcTub (green). (A) *min1 mlt1* double-mutant cell with an ultramini eyespot in the anterior lobe of the chloroplast. (B) *min2 mlt1* cell with ultramini eyespot at the anterior of the chloroplast lobe. (C) *min1 mlt1* cell with a small, anterior, asymmetric ChR1 spot. (D) *min2 mlt1* cell with a small, asymmetric ChR1 spot associated with a short rootlet. (E) *min1 mlt2* double-mutant cell with four eyespots, exhibiting the *mlt2* phenotype. Combined image from two focal planes. (F) *min2 mlt2* double-mutant cell with three eyespots, exhibiting the *mlt2* phenotype. (G) *min1 mlt2* cells. Cell at bottom exemplifies the clustering of ChR1 spots often seen around the anterior pole in both multiple-eyespot mutants. (H) *min2 mlt2* cell with multiple photoreceptor patches. Scale bars, 5μm.

To further characterize the effects of eyespot mutations on assembly of the organelle, eyespots were examined in double-mutant combinations of mini- and multiple-eyespot mutants. Of eyespots scored by bright field microscopy in a population of *min2 mlt1* double-mutant cells (n = 244), 76% had one eyespot, 13% had two eyespots, and 11% had no observable eyespot, in sharp contrast to the mostly multiple-eyespot phenotype of *mlt1* cells after the same growth period (Table 2). Eyespots of double-mutant cells ranged in size from ultraminiature to approximately *min2*-sized, and ranged in position from the anterior tip of the chloroplast lobe (Figure 7B) to an approximately equatorial position. Among *min2 mlt1* cells with two eyespots, the proportion of eyespots on the same *vs.* opposite sides of the cell did not differ substantially from the proportions observed in *mlt1* populations (Table 4). These data imply that the *min2* mutation suppresses the eyespot number defect of *mlt1*, but it does not alter the defect in eyespot placement.

**Table 4:** Eyespot phenotypes of *min2 mlt1* and *min1 min2* double mutants and *mlt1* cells

Strain	Cells Scored	Eyespots/Cell (%)					Position of Multiple Eyespots in Cell*^a^*	
		0	1	2	3	4-5	Same	Opposite
*min1 min2*	101	59 (58%)	42 (42%)	0	0	0	N/A	
*min2 mlt1*	244	27 (11%)	185 (76%)	32 (13%)	0	0	19 (59%)	13 (41%)
*mlt1* log phase	107	0	15 (14%)	80 (75%)	7 (7%)	5 (5%)	40 (46%)	47 (54%)
*mlt1* stationary phase	104	0	0	35 (34%)	56 (54%)	13 (12%)	42 (46%)	49 (54%)

a Excludes *mlt1* cells with four or more eyespots.

**Figure 8:**
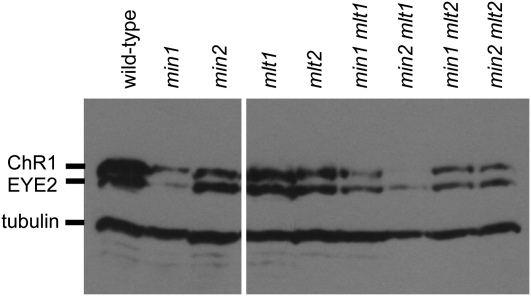
Immunoblot of wild-type and mutant strains probed for ChR1, EYE2, and tubulin. Whole-cell extracts were prepared after overnight growth in M medium. ChR1 and EYE2 levels were reduced approximately 75% and 93%, respectively, in *min1* and 53% and 32%, respectively, in *min2* single mutants compared with wild-type. ChR1 and EYE2 levels were reduced by approximately 75% and 65%, respectively, in the *min1 mlt1* double mutant and approximately 99% and 85%, respectively, in the *min2 mlt1* double mutant compared with wild-type. ChR1 and EYE2 were reduced 43% and 41%, respectively, in the *mlt2* single mutant compared with wild-type, whereas ChR1 and EYE2 were reduced 66% and 62%, respectively, in *min1 mlt2* and 77% and 62%, respectively, in *min2 mlt2* compared with wild-type.

The combination of the *min1* mutation with *mlt1* produces a synthetic phenotype in which double-mutant cells are either eyeless or possess a single ultraminiature spot of unorganized pigment granules at or near the anterior tip of the chloroplast lobe (Lamb *et al.* 1999; Figure 7A). ChR1 localization in the *min1 mlt1* double mutant mirrored eyespot position as observed by bright field microcopy, appearing as a single, small spot near the anterior of the cell (Figure 7C). A similar phenotype was observed in some *min2 mlt1* cells, with a small ChR1 spot appearing at or near the anterior of the cell (Figure 7D). However, most *min2 mlt1* cells had no observable ChR1 staining, paralleled in immunoblot analysis by drastically reduced overall ChR1 levels compared with wild-type and either single mutant (Figure 8). Mutation of either miniature-eyespot locus in the *mlt1* background thus results in magnified attenuation of ChR1 expression or stability when compared with the reduction observed in either the *min1* or *min2* single mutants.

### Eyespot phenotype of *mlt2* is epistatic to that of *min1* and *min2*

Interestingly, combinations of *mlt2* with either *min1* or *min2* did not yield synthetic eyespot phenotypes similar to those observed in *min1 mlt1* or *min2 mlt1*. The *min1 mlt2* or *min2 mlt2* combination resulted in cells that exhibited the multiple-eyespot phenotype of *mlt2*, demonstrative that the multiple-eyespot phenotype of *mlt2* is epistatic to both miniature-eyespot mutations (Figure 7, E and F). Although the overall levels of ChR1 and EYE2 were reduced in the *min1 mlt2* and *min2 mlt2* strains compared with the *mlt2* single mutant (Figure 8), eyespot sizes in the *min1 mlt2* and *min2 mlt2* double mutants were not noticeably smaller than the range of sizes observed in *mlt2* alone, and the localization patterns of ChR1 in *min1 mlt2* and *min2 mlt2* cells were similar to those observed in *mlt2* cells (Figure 7, G and H). Thus, the *min1* and *min2* mutations affect steady-state levels of eyespot proteins but do not alter localization of photoreceptor to multiple patches in the *mlt2* mutant background. The extremely low recombination frequency of *mlt1* with *mlt2* precluded isolation of multiple strains to confirm the phenotype of *mlt1 mlt2* double mutants, but phenotypic analysis of progeny from a tetratype tetrad from a *mlt1* × *mlt2* cross was suggestive that *mlt2* is also epistatic to *mlt1* with regard to the number of supernumerary eyespots. Examination of diploids heterozygous for the either the *mlt1* or *mlt2* mutation indicated that neither mutation was dominant (10 ptx^+^ diploids, n = 10). These analyses are suggestive that MLT2 regulates eyespot number via a mechanism distinct from that of MLT1.

### *MIN2* and *MLT2* loci map to existing eyespot gene clusters

The previously described *MIN1*, *MLT1*, and *EYE2* loci were found to be mutually linked on chromosome 12 (Lamb *et al.* 1999), whereas the *EYE3* locus is unlinked to the other three loci and maps to chromosome 2. Surprisingly, the *MLT2* locus is very tightly linked to *MLT1*, with an estimated genetic distance of 0.50 map units, which is approximately equivalent to 50 kbp (Table 5). We are at present uncertain of the position of *MLT2* relative to *EYE2*. Analysis of tetrad products from crosses of *min2* to an allele of *eye3* revealed that the *min2* mutation is linked to *eye3* within 7 map units (Table 5). The *min2-eye3* linkage on chromosome 2 thus constitutes a second cluster of eyespot-assembly loci in *C. reinhardtii* in addition to that on chromosome 12.

**Table 5:** Linkage data for eyespot-assembly loci

Cross	PD:NPD:TT	Total	Recombination Frequency	Estimated Map Units
*mlt1* × *mlt2*	121: 0: 1	122	0.005	0.50
*min1* × *mlt2*	92: 4: 12	108	0.093	9.3
*eye3* × *min2*	119: 0: 18	137	0.066	6.6

## Discussion

The complex structure of the photosensory eyespot organelle of *Chlamydomonas reinhardtii* has invited investigation of the factors that contribute to the organization of this system from multiple cellular compartments. Several phototaxis-negative mutants with defects in eyespot assembly and placement have been isolated and have been informative in contributing to our understanding of the biogenesis and asymmetric positioning of this organelle (Dieckmann 2003; Kreimer 2009). Eyespot-assembly mutants fall into three main classes: eyeless mutants lacking organized pigment granule stacks, strains with a miniature eyespot, and strains possessing multiple eyespots. In this study, the miniature-eyed mutant *min2* and multiple-eyespot mutant *mlt2* have been further characterized and their loci mapped. Effects of these mutations on eyespot organization and steady-state levels of eyespot proteins have been assessed and are discussed in light of current understanding of eyespot biogenesis.

### Miniature-eyespot *MIN2* locus affects photoreceptor level and size, but not overall organization, of the eyespot

Like the *min1* mutant strain, cells of the *min2-1* strain have a miniature eyespot located at the equator of the cell; in contrast to *min1*; however, the eyespot in *min2* cells exhibited a properly layered morphology of photoreceptor and pigment granules. *min2* cells are unable to phototax but can swim away from high-intensity light, although to a lesser extent than either *min1* or the eyeless strains (Roberts 1999). These data were suggestive that the *min2* mutation affects some aspect of the signal transduction pathway downstream of photoreceptor activation (Roberts 1999). The MIN2 protein may modulate the capacity of the photoreceptors for transduction of the photosignal upon light activation. Interestingly, mutation of the *MIN2* locus in the *mlt1* mutant background results in nearly complete abolition of ChR1 photoreceptor levels. MIN2 may be conditionally required for stability of photoreceptor molecules at a stage of the eyespot assembly process in conjunction with MLT1 function, whereas it is not required for photoreceptor stability in the organized eyespot layers. However, the enhanced eyespot-assembly defect of *min1 min2* double mutants may point to some role of MIN2 in promotion of structural stability of eyespot components.

### *MLT2* locus regulates eyespot number and is required for proper eyespot organization and placement

Both multiple-eyespot mutant strains, *mlt1* and *mlt2*, are characterized by supernumerary, nonasymmetrically localized eyespots. The phenotype of the *mlt2* mutant is highly similar to that of the multiple-eyespot mutant *mes-10*, described by Nakamura *et al.* (2001). It is likely that the *mes-10* mutation is allelic to *mlt2-1*, but as *mes-10* gametes are unable to mate (Nakamura *et al.* 2001), it was not possible to carry out genetic characterization of this mutant strain. Like *mlt2* cells, *mes-10* cells have one to over four eyespots per cell that display a range of sizes, and half of *mes-10* cells possessed two pyrenoids, a phenotype also observed in *mlt2*, albeit at a lower frequency. The ultrastructure of *mes-10* eyespots examined by electron microscopy appeared normal, with regular pigment granule layers and the chloroplast envelope apposed to the plasma membrane, although occasional pigment granule stacks faced the surface of the chloroplast envelope near the nucleus rather than being apposed to the plasma membrane.

A high proportion of spots wherein EYE2, EYE3, and ChR1 are mutually copositioned was observed during logarithmic phase in *mlt1* and *mlt2* cells, suggestive that eyespots in these mutants initially form according to the normal assembly process and subsequently lose structural integrity. Thus, the appearance of multiple spots of eyespot components in distinct and often widely separated punctae in stationary-phase cells likely represents eyespots at various stages of disintegration. In both *mlt1* and *mlt2* cells, pigment granules and photoreceptors were never observed without EYE2, demonstrating that the EYE2 patch, which is likely anchored to the chloroplast envelope, is required for linkage of the plasma membrane–localized photoreceptors to the pigment granule layers of the eyespot.

The defects in the placement of eyespot compartments observed in both the *mlt1* and *mlt2* mutants indicate that upregulation of eyespot number disrupts maintenance of eyespot organization. Further, the observation of multiple pyrenoids in a substantial subset of *mlt2* cells intimates that the roles of MLT2 extend beyond eyespot formation to processes affecting the biogenesis of other chloroplastic structures. MLT2 may be a transcriptional or translational repressor under cell-cycle control, wherein loss of function leads to continued synthesis of assembly proteins beyond the typical window of eyespot development. The phenotypes of miniature- and multiple-eyespot mutants are consistent with the idea that eyespot assembly follows a “quantal synthesis” model of organelle biogenesis (Stephens 1989; Rafelski and Marshall 2008), in which lack of some eyespot proteins results in a smaller organelle (as in the *min1* and *min2* mutants), whereas misregulated expression of other eyespot components leads to the formation of multiple eyespots that lose structural integrity if other critical proteins are limiting. Application of this model has been demonstrated experimentally in studies on *C. reinhardtii* flagella (Rosenbaum *et al.* 1969; Coyne and Rosenbaum 1970). It would be informative to follow expression of eyespot-assembly genes during the cell cycle in multiple-eyespot mutants.

### Miniature-eyespot loci suppress the multiple-eyespot phenotype of *mlt1*

In combination with *mlt1*, both the *min1* and *min2* mutations suppress the multiple-eyespot phenotype of *mlt1*. It is possible that limitation of eyespot material caused by absence of MIN1 or MIN2 masks the disregulation of eyespot localization and number cues resulting from mutations in *MLT1*. The epistasis of *mlt2* to *min1* and *min2*, in addition to the ability of *mlt2* cells to assemble wild-type–sized eyespots, may indicate that the upregulation of eyespot number in the *mlt2* mutant overcomes limitations imposed by the relatively lower abundance of some eyespot proteins. The dramatic differences between combinations of miniature-eyespot mutations with *mlt1* and *mlt2* reinforce the hypothesis that the mode of regulation of eyespot number and placement by *MLT1* is distinct from that of *MLT2*. Characterization and localization of the MIN and MLT proteins should greatly assist our understanding of the mechanisms of action of these factors.

It is quite interesting that both the *min2* and *mlt2* mutations map to novel loci near existing eyespot-assembly genes. As suggested in Lamb *et al.* (1999), the clustering of eyespot-assembly loci may be an instrument for coordinated gene expression that is linked to cell-cycle control. The tight linkage, especially of *mlt2* to *mlt1*, is supportive of the idea that the *MLT1* and *MLT2* genes, though distinct, affect similar aspects of eyespot development and are coordinately regulated. Identifying the gene products of these loci and determining the nature of the *min2* and *mlt2* mutations will be valuable for extending our knowledge of the assembly and organization of this intriguing organelle.

## Supplementary Material

Supporting Information
