# Supplementary material for: Miniature- and Multiple-Eyespot Loci in Chlamydomonas reinhardtii Define New Modulators of Eyespot Photoreception and Assembly
Source: G3 (Bethesda). 2011 Nov 1;1(6):489–98. doi: 10.1534/g3.111.000679 (PMC3276157; doi:10.1534/g3.111.000679)
Supplement: Supporting Information [file supp_1_6_489__index.html]

Supporting Information 

# Miniature- and Multiple-Eyespot Loci in *Chlamydomonas reinhardtii* Define New Modulators of Eyespot Photoreception and Assembly

## Supporting Information for Boyd, Lamb, and Dieckmann, 2011

**Files in this Data Supplement:**

- Supporting Information - Figure S1 and Table S1 (PDF, 152 KB)
- Figure S1 - Comparison of growth rates of wild-type and *mlt2* (PDF, 104 KB)
- Table S1 - Normalized average growth rates of wild-type and *mlt2* strains (� S. D.) (PDF, 52 KB)
