# Supplementary material for: Miniature- and Multiple-Eyespot Loci in Chlamydomonas reinhardtii Define New Modulators of Eyespot Photoreception and Assembly
Source: G3 (Bethesda). 2011 Nov 1;1(6):489–98. doi: 10.1534/g3.111.000679 (PMC3276157; doi:10.1534/g3.111.000679)
Supplement: Supporting Information [file supp_1.6.489_000679SI.pdf]

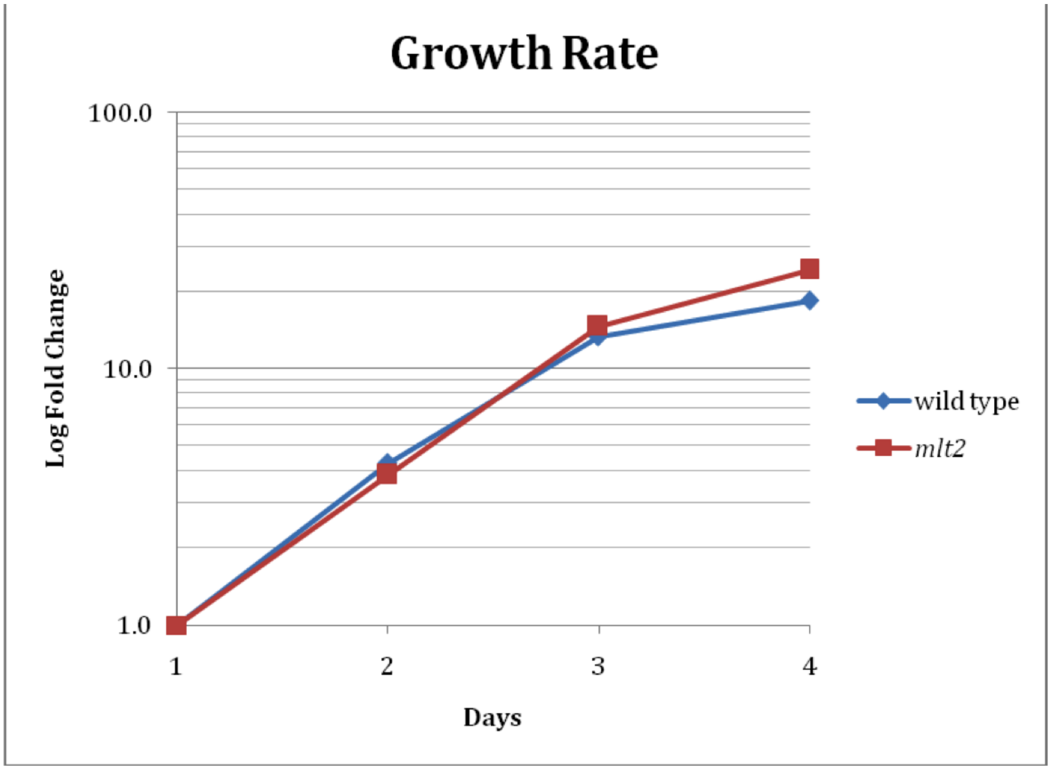


**Figure S1** Comparison of growth rates of wild-type and *mlt2*. Log-fold change in cell density was measured over four days and each strain was normalized to Day 1 measurements. The growth rate of *mlt2* did not differ significantly from wild-type.

**Table S1 Normalized average growth rates of wild-type and *mlt2* strains (± S. D.).** The growth rate of the *mlt2* strain over 4 days did not differ significantly from wild-type.

| Strain | Fold Change | | | |
|---|---|---|---|---|
| | Day 1 | Day 2 | Day 3 | Day 4 |
| wild-type | 1 | 4 ±1 | 13±4 | 18 ±6 |
| *mlt2* | 1 | 3.9±0.7 | 15±3 | 24±4 |
| wild-type vs. *mlt2* P(0.05) | -- | 0.31 | 0.33 | 0.09 |
