# Supplementary material for: Miniature- and Multiple-Eyespot Loci in Chlamydomonas reinhardtii Define New Modulators of Eyespot Photoreception and Assembly
Source: G3 (Bethesda). 2011 Nov 1;1(6):489–98. doi: 10.1534/g3.111.000679 (PMC3276157; doi:10.1534/g3.111.000679)
Supplement: Supporting Information [file supp_1.6.489_FigureS1.pdf]

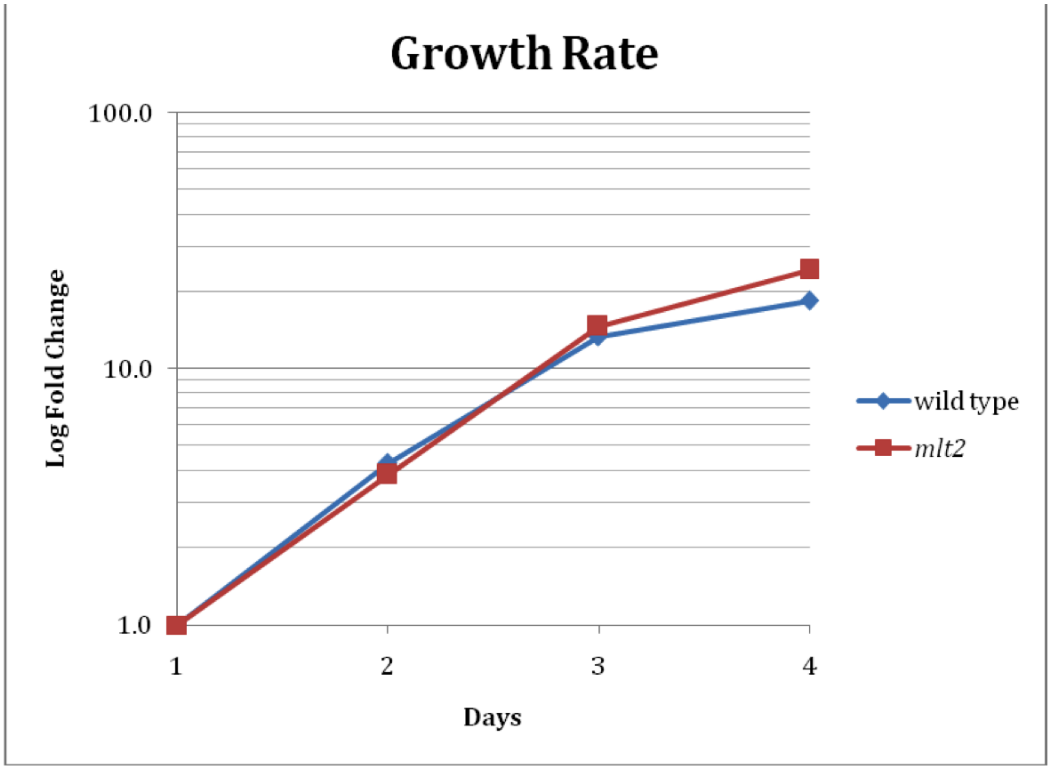

Figure S1 Comparison of growth rates of wild-type and *mlt2*. Log-fold change in cell density was measured over four days and each strain was normalized to Day 1 measurements. The growth rate of *mlt2* did not differ significantly from wild-type.
