# Supplementary material for: Miniature- and Multiple-Eyespot Loci in Chlamydomonas reinhardtii Define New Modulators of Eyespot Photoreception and Assembly
Source: G3 (Bethesda). 2011 Nov 1;1(6):489–98. doi: 10.1534/g3.111.000679 (PMC3276157; doi:10.1534/g3.111.000679)
Supplement: Supporting Information [file supp_1.6.489_TableS1.pdf]

Table S1 Normalized average growth rates of wild-type and *mlt2* strains (± S. D.). The growth rate of the *mlt2* strain over 4 days did not differ significantly from wild-type.

Strain	Fold Change			
	Day 1	Day 2	Day 3	Day 4
wild-type	1	4 ±1	13±4	18 ±6
mlt2	1	3.9±0.7	15±3	24±4
wild-type vs. *mlt2* P(0.05)	--	0.31	0.33	0.09
